# Fatal Association of Eisenmenger Syndrome and Severe Preeclampsia

**DOI:** 10.7759/cureus.37836

**Published:** 2023-04-19

**Authors:** Said Benlamkaddem, Fatima Bouyermane, Djoudline Doughmi, Mohamed Adnane Berdai, Mustapha Harandou

**Affiliations:** 1 Maternal and Pediatric Critical Care Unit, Hassan II University Hospital, Fez, MAR; 2 Faculty of Medicine and Pharmacy, Sidi Mohamed Ben Abdellah University, Fez, MAR

**Keywords:** pulmonary hypertension, regional anesthesia, general anesthesia, severe preeclampsia, eisenmenger syndrome

## Abstract

Eisenmenger syndrome (ES) is the end stage of pulmonary arterial hypertension (PAH) associated with congenital heart disease (CHD), which can occur in patients with large, unrepaired cardiac shunts (ventricular septal defects (VSD), atrial septal defects (ASD), and patent ductus arteriosus (PDA)). Pregnancy in Eisenmenger syndrome is uncommon and is poorly tolerated due to physiological changes that may lead to a risk of rapidly progressive cardiopulmonary decompensation, thrombotic complications, and sudden death. For these reasons, it is advisable, in this context, to avoid pregnancy or to undergo an early pregnancy termination within the tenth gestational week. The occurrence of severe preeclampsia in this situation leads to fatal maternal and fetal outcomes.

We report the case of a 23-year-old female patient, gravida 1 nullipara at the thirty-fourth week of gestation, with a history of a persistent ductus arteriosus (PDA) in childhood that progressed to ES. She was admitted to the obstetric emergency for respiratory distress associated with signs of low cardiac output. CT pulmonary angiography and transthoracic echocardiography showed no pulmonary embolism, an enlarged pulmonary artery, dilated right cardiac chambers (ventricle and atrium) compressing the left ones, a right ventricular/left ventricular (RV/LV) ratio > 1, a persistent ductus arteriosus, and a calculated systolic pulmonary arterial pressure (PAPS) at 130 mmHg. She also had severe preeclampsia with evolutive HELLP (hemolysis, elevated liver enzymes, low platelet count) syndrome and intrauterine fetal death indicating fetal delivery under general anesthesia after platelets transfusion. At the end of the surgery, the patient presented a sudden death following a cardiac arrest despite 45 minutes of cardiopulmonary resuscitation.

## Introduction

Eisenmenger syndrome (ES) is the end stage of pulmonary arterial hypertension (PAH) associated with congenital heart disease (CHD), which can occur in patients with large, unrepaired intra or extracardiac shunts (ventricular septal defects (VSD), atrial septal defects (ASD), and patent ductus arteriosus (PDA)) [1]. Pregnancy in Eisenmenger syndrome is uncommon and is poorly tolerated due to physiological changes that may lead to a high risk of rapidly progressive cardiopulmonary decompensation, thrombotic complications, and sudden death [2]. For these reasons, it is advisable, in this context, to avoid pregnancy or to undergo an early pregnancy interruption within the tenth gestational week [3].

Severe preeclampsia, which complicates 2% to 4% of pregnancies, is considered the second cause of maternal deaths and perinatal complications worldwide [4,5].

We report a case of the association between severe preeclampsia in a 23-year-old female gravida 1 nullipara at the thirty-fourth week of gestation with Eisenmenger syndrome, which led to maternal and fetal fatal outcomes.

## Case presentation

**Figure 1:**
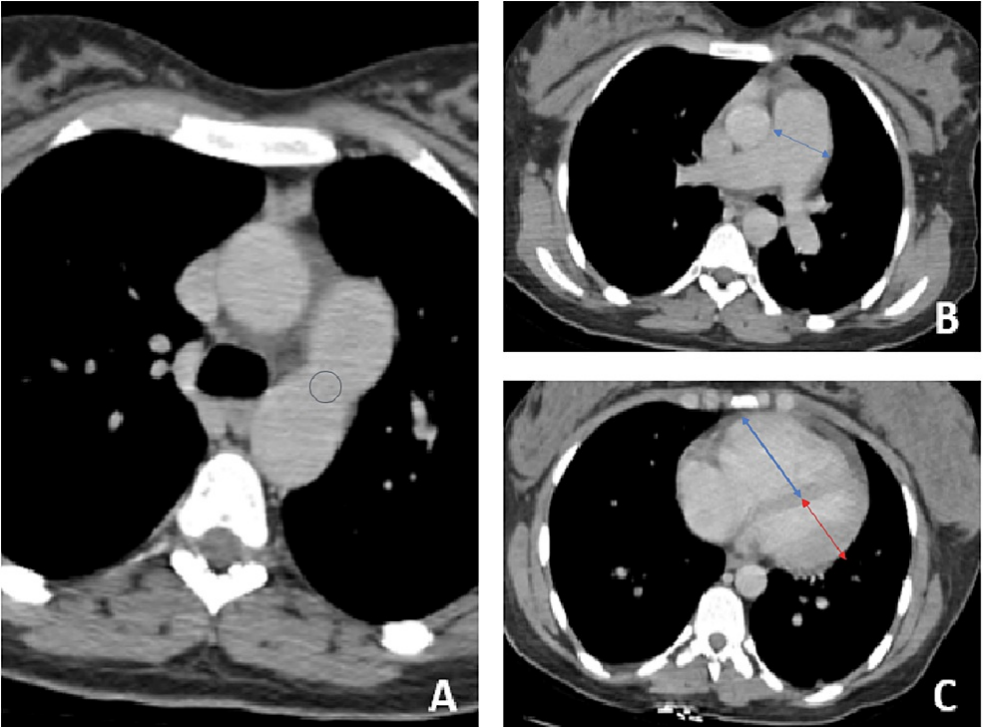
CT pulmonary angiography showed a persistent ductus arteriosus (A, circle), an enlarged pulmonary artery (B, blue arrow), and dilated right ventricle (blue arrow) compressing the left ventricle (red arrow) (C)

We report a case of a 23-year-old female patient, gravida 1 nullipara at her thirty-fourth week of gestation, with a history of persistent ductus arteriosus (PDA) since childhood. She underwent catheterization at the age of eight, which concluded in Eisenmenger syndrome contraindicating the closure of PDA. Since that time the patient was lost to follow-up. She was admitted to the obstetric emergency department for respiratory distress. At her first clinical examination, she was confused with profuse sweating, had cold and cyanotic extremities, polypnea at 40 breaths/min, normal lung auscultation, oxygen desaturation (SpO2 at 30%), blood pressure at 90/60mmHg, tachycardia at 120 beats/min, and positive proteinuria at urine dipstick. Immediately, the patient was intubated following a rapid sequence induction (RSI) using propofol, rocuronium, and fentanyl. Norepinephrine was started through a right internal jugular central line. Despite ventilation with the fraction of inspired oxygen (FiO2) at 1, oxygen saturation (SpO2) remained low. CT pulmonary angiography showed no pulmonary embolism, an enlarged pulmonary artery, dilated right cardiac chambers (ventricle and atrium) compressing the left ones, a right ventricular/left ventricular (RV/LV) ratio > 1, and a persistent ductus arteriosus (Figure 1).

The transthoracic echocardiography confirmed CT scan findings with a calculated systolic pulmonary arterial pressure (PAPS) at 130 mmHg. No intracardiac shunts were found. Inhaled nitric oxide (iNO) was then initiated at a dose of 40 ppm, resulting in a spectacular respiratory and hemodynamic improvement allowing to decrease the FiO2 to 0.5 and to halt the norepinephrine.

The obstetric ultrasound objectified an intrauterine fetal death. Laboratory tests showed hemoglobin at 13.4g/dl, thrombocytopenia at 9000/mm³, prothrombin time (PT) at 100%, lactate dehydrogenase (LDH) at 879 IU/ml, hypoalbuminemia at 25g/l, normal liver, and renal function (Table 1).

**Table 1:** Laboratory findings at admission ASAT: aspartate aminotransferase; ALAT: alanine aminotransferase; LDH: lactate dehydrogenase; BUN: blood urea nitrogen

Parameters	Admission	Normal range
Hemoglobin (g/dL)	13.4	11.5-15
Platelets (x10^3^ /µL)	9	150-450
Prothrombin Time (PT%)	100	> 70
ASAT (UI/L)	32	0-50
ALAT (IU/L)	13	0-50
LDH (IU/L)	879	0-240
BUN (g/L)	0.3	0.1- 0.4
Serum creatinine (mg/L)	8.7	5-13
Albumin (g/L)	25	35-52

Based on the laboratory findings mentioned earlier and the patient becoming hypertensive at 170/110 mmHg, the diagnosis of severe pre-eclampsia with evolutive HELLP (hemolysis, elevated liver enzymes, low platelet count) syndrome was retained, indicating the delivery of the fetus immediately after platelets transfusion, through cesarean section under general anesthesia. During the surgery, the patient was lung-protective ventilated and on continuous inhaled nitric oxide (iNO). She received fluid boluses of 250 ml and a bolus of diluted norepinephrine to maintain high blood pressure levels. The maintenance of anesthesia was achieved with intravenous agents (propofol and fentanyl). A 10-minute infusion of oxytocin to prevent uterine atony was given after placenta delivery. At the end of the surgery, the patient presented sudden death following a cardiac arrest despite 45 minutes of cardiopulmonary resuscitation.

## Discussion

Eisenmenger’s syndrome (ES) was first described in 1897 and was later identified as the end stage of PAH associated with a large and unrepaired intracardiac or extracardiac connection between systemic and pulmonary circulation [1,6]. In pregnancy, ES is rare and is characterized by a high risk of maternal and fetal morbidity and mortality ranging between 26% and 66% despite advanced obstetric care [7]. The association between ES and preeclampsia, which is a severe obstetric complication, can be challenging. As a result, patients should be counseled against pregnancy. If pregnancy occurs, therapeutic abortion is the proper management. If the patient decides to continue the pregnancy, a close follow-up should be planned [8].

Pregnancy in ES is a situation of cardiac stress due to the hemodynamic changes it induces. Indeed, there is a decrease in systemic vascular resistance in response to hormonal changes (beta HCG, progesterone, and estrogen) and an increased cardiac output due to an increase in both heart rate and stroke volume [9]. Severe preeclampsia in this context may induce more adverse effects due to inflammation and endothelial dysfunction leading to increased vascular and pulmonary vascular resistance and altered diastolic ventricular function [10]. As a result, the preload reserve function of the right heart is significantly reduced making unbearable any fluid changes [11].

The ideal mode of delivery is controversial. Labor and delivery are associated with an increase in venous return because of uterine contraction, which releases up to 500 ml of blood into the circulation, and the decompression of the aorta and vena cava. Epidural analgesia may attenuate this effect [3,9]. Despite the high risk of morbidity (blood loss, thrombosis, infection, pain, etc.), elective cesarean section is preferred by different teams because it allows the presence of senior multidisciplinary staff at a time of optimal circumstances, and makes performing tubal sterilization possible (if the patient wishes) to prevent further pregnancy [9,12]. Cesarean section in our case was inevitable because of the severity of PAH and the presence of severe preeclampsia with evolutive HELLP syndrome.

Anesthetic management of patients with ES is challenging. General anesthesia may have a negative effect. Most anesthetic drugs reduce systemic vascular resistance, which may increase right to left shunt and reduce cardiac output. Positive pressure mechanical ventilation increases the right ventricle afterload and thus reduces cardiac output by compression of the left ventricle. Hemodynamic monitoring with careful choice of anesthetic drugs, ketamine and etomidate, may be of interest in this indication; the use of lung protective ventilation and vasopressors are judicious actions to offset the negative impact of general anesthesia [3,9,11]. Single-shot spinal anesthesia with peripheral vasodilation should be avoided due to the risks of potentially severe and life-threatening hypotension in the setting of severe right ventricular impairment. Epidural anesthesia or a combined spinal-epidural approach is well-tolerated hemodynamically. The latter approach provides the advantages of a low spinal block with a good somatic and visceral analgesia than an epidural, whilst avoiding the risk of hypotension with a spinal. Regional anesthesia also has the advantage of providing postoperative analgesia [3,9,13]. The choice of anesthesia technique for our patient was not feasible, as she was urgently intubated at her admission regarding the severity of the respiratory distress, and the HELLP syndrome with severe thrombocytopenia contraindicating regional anesthesia.

The postpartum period is considered very risky with mortality as the maximum, as all hemodynamic changes begin to resolve. Indeed, the change in hormone level, the release of abdominal pressure as well as the shift of fluid of tissue edema in severe preeclampsia to the circulation increase venous return leading to right ventricle failure [7,11,14].

Our patient's cardiac arrest was a result of a conjunction of multiple factors. In addition to all pregnancy-related hemodynamic changes, she had been lost to follow-up for her congenital cardiac disease and admitted, already pregnant at the thirty-fourth week of gestation in a severe clinical picture. The presence of severe preeclampsia with HELLP syndrome and fetal death worsened the outcome.

## Conclusions

Eisenmenger syndrome and pregnancy is an uncommon association characterized by a high risk of maternal and fetal mortality. Preeclampsia, which is a severe complication of pregnancy, worsens the outcome. Despite advanced obstetric care, the European Society of Cardiology recommends counseling against pregnancy, and if so, discussing the termination of pregnancy as soon as possible. If the patient wishes to continue the pregnancy, close monitoring by a multidisciplinary team, including obstetricians, cardiologists, and anesthesiologists is necessary.

## References

[1] Wood P (1958). The Eisenmenger syndrome or pulmonary hypertension with reversed central shunt. Br Med J.

[2] Lopez BM, Malhamé I, Davies LK, Gonzalez Velez JM, Marelli A, Rabai F (2020). Eisenmenger syndrome in pregnancy: a management conundrum. J Cardiothorac Vasc Anesth.

[3] Yuan SM (2016). Eisenmenger syndrome in pregnancy. Braz J Cardiovasc Surg.

[4] Magee LA, Nicolaides KH, von Dadelszen P (2022). Preeclampsia. N Engl J Med.

[5] Phupong V, Ultchaswadi P, Charakorn C, Prammanee K, Prasertsri S, Charuluxananan S (2003). Fatal maternal outcome of a parturient with Eisenmenger's syndrome and severe preeclampsia. Arch Gynecol Obstet.

[6] Duke M (2017). Victor Eisenmenger (1864-1932): the man behind the syndrome. J Med Biogr.

[7] Low TT, Guron N, Ducas R, Yamamura K, Charla P, Granton J, Silversides CK (2021). Pulmonary arterial hypertension in pregnancy—a systematic review of outcomes in the modern era. Pulm Circ.

[8] Regitz-Zagrosek V, Roos-Hesselink JW, Bauersachs J (2018). 2018 ESC Guidelines for the management of cardiovascular diseases during pregnancy. Eur Heart J.

[9] ten Klooster L, Wilson VJ, Newton R, Selby K, Gandhi SV, Kiely DG (2020). Pulmonary hypertension and pregnancy. Respiratory Disease in Pregnancy.

[10] Melchiorre K, Sharma R, Thilaganathan B (2014). Cardiovascular implications in preeclampsia: an overview. Circulation.

[11] Chen Z, Liu Y, Tan G, You D, Sun X (2020). When severe preeclampsia met pulmonary arterial hypertension: if time goes back. Authorea.

[12] Slaibi A, Ibraheem B, Mohanna F (2021). Challenging management of a pregnancy complicated by Eisenmenger syndrome; a case report. Ann Med Surg (Lond).

[13] Rathod S, Samal SK (2014). Successful pregnancy outcome in a case of Eisenmenger syndrome: a rare case report. J Clin Diagn Res.

[14] Vashisht A, Katakam N, Kausar S, Patel N, Stratton J (2015). Postnatal diagnosis of maternal congenital heart disease: missed opportunities. BMJ Case Rep.

